# Predicting occupational injury causal factors using text-based analytics: A systematic review

**DOI:** 10.3389/fpubh.2022.984099

**Published:** 2022-09-15

**Authors:** Mohamed Zul Fadhli Khairuddin, Khairunnisa Hasikin, Nasrul Anuar Abd Razak, Khin Wee Lai, Mohd Zamri Osman, Muhammet Fatih Aslan, Kadir Sabanci, Muhammad Mokhzaini Azizan, Suresh Chandra Satapathy, Xiang Wu

**Affiliations:** ^1^Department of Biomedical Engineering, Faculty of Engineering, Universiti Malaya, Kuala Lumpur, Malaysia; ^2^Institute of Medical Science Technology, Universiti Kuala Lumpur, Selangor, Malaysia; ^3^Centre of Intelligent Systems for Emerging Technology (CISET), Faculty of Engineering, Universiti Malaya, Kuala Lumpur, Malaysia; ^4^Faculty of Computing, College of Computing and Applied Science, Universiti Malaysia Pahang, Gambang, Malaysia; ^5^Department of Electrical and Electronics Engineering, Karamanoglu Mehmetbey University, Karaman, Turkey; ^6^Department of Electrical and Electronic Engineering, Faculty of Engineering and Built Environment, Universiti Sains Islam Malaysia, Nilai, Negeri Sembilan, Malaysia; ^7^School of Computer Engineering, Kalinga Institute of Industrial Technology, Deemed to Be University, Bhubaneswar, India; ^8^School of Medical Information and Engineering, Xuzhou Medical University Xuzhou, Xuzhou, Jiangsu, China

**Keywords:** natural language processing, artificial intelligence, machine learning, deep learning, occupational health and safety

## Abstract

Workplace accidents can cause a catastrophic loss to the company including human injuries and fatalities. Occupational injury reports may provide a detailed description of how the incidents occurred. Thus, the narrative is a useful information to extract, classify and analyze occupational injury. This study provides a systematic review of text mining and Natural Language Processing (NLP) applications to extract text narratives from occupational injury reports. A systematic search was conducted through multiple databases including Scopus, PubMed, and Science Direct. Only original studies that examined the application of machine and deep learning-based Natural Language Processing models for occupational injury analysis were incorporated in this study. A total of 27, out of 210 articles were reviewed in this study by adopting the Preferred Reporting Items for Systematic Review (PRISMA). This review highlighted that various machine and deep learning-based NLP models such as K-means, Naïve Bayes, Support Vector Machine, Decision Tree, and K-Nearest Neighbors were applied to predict occupational injury. On top of these models, deep neural networks are also included in classifying the type of accidents and identifying the causal factors. However, there is a paucity in using the deep learning models in extracting the occupational injury reports. This is due to these techniques are pretty much very recent and making inroads into decision-making in occupational safety and health as a whole. Despite that, this paper believed that there is a huge and promising potential to explore the application of NLP and text-based analytics in this occupational injury research field. Therefore, the improvement of data balancing techniques and the development of an automated decision-making support system for occupational injury by applying the deep learning-based NLP models are the recommendations given for future research.

## Introduction

International Labor Organization (ILO) defined occupational injury as “any personal injury, disease or death resulting from an occupational accident” (1). The accidents may arise out of or in connection with tasks resulting in workers incurring injury, disease, or fatality. An occupational injury could be fatal or non-fatal. Nearly half of the workers in highly industrialized countries and developing countries are exposed to the risk of fatal injuries (2). World Health Organization (WHO) estimated that nearly 374 million occupational injuries are reported annually, resulting in more than 2.3 million annual fatalities each year worldwide (3).

Occupational injuries can affect workers' lives and may cause a substantial economic burden to employees, employers, and society (3, 4). In terms of economic impact, it affects the organizational performance such as the indirect costs of the workplace to the employer and employees, especially on the workers' compensation for lost earnings, and medical and rehabilitation expenses (5). Nurul Ayuni et al. in their study concluded that the effects of occupational accidents include loss of ability to work which may affect individual or family income and alteration of living standard. Furthermore, disruptive changes in the workers' psychological and behavioral state may cause other related health problems such as traumatized experiences and insecurity feeling during work. This will consequently disrupt work performance, delay work progress, loss of time in project execution, and loss of productivity (6).

In addition, Azizah et al. argued that occupational injuries may have a major impact on the company's financial performance. This is due to additional expenses such as damage, medical, and legal cost, not to mention lower output due to stopped operation, and loss of personal and working time (7). In a recent study by Kim and Park, it was found that the increment of occupational accidents reduced sales per employee, operating profit per employee, the ratio of operating profit to sales, and sales growth rate by a statistically significant level (8). Therefore, from the trends of the published works discovered, we can observe continuous decrements in institutional performances caused by the severity of occupational injuries.

Hence, intensive research on occupational injury is vital to alleviate the existence of accidents at the workplace especially in extracting information from occupational injury reports. Previous trends in occupational injury analysis were developed from a general descriptive statistical analysis (9), decision trees (10), generalized linear model (11), and fuzzy-neural method (12) that have all been used to analyze the accident and injury data to reduce the injury rates. To add, Papazoglou et al. used Bayesian network techniques to quantify the occupational injury rates (13), meanwhile, Yorio et al. utilized Poisson models for occupational injury risk assessments (14).

Though, a significant problem with these existing models is their limited ability to process large-scale raw data (11). Besides, these techniques simplify some key factors and pay little attention to analyzing the linkages between a safety phenomenon such as occupational accidents and the safety data (15).

The reports on occupational health and safety are necessary and sought after for maintenance purposes, from the perspective of the reporting requirements. However, as the events passed, the reports are often deemed insignificant, with no following up actions. While businesses progressed better in the aspect of prevention, most of safety indicators remain retrospective. Despite organizations have experienced fatal, serious, and lost time events, they seemed to ignore these signals and do not learn from them. With such huge historical data at hand, together with proper strategy and know-how, the organizations should equip themselves with forecasting capability and be able to foresee future occurrences of such events. This could be the new added value to the related ecosystem and industries, whereas the risk of occupational injuries is anticipated, and therefore managed efficiently.

Therefore, leveraging the big data technology while assessing occupational safety and health risks is deemed necessary. It is an emerging technology due to its parallel processing feature and ability to efficiently handle high-dimensional data. Big data is crucial especially in exploratory, descriptive, predictive and prescriptive to determine future trends or events (16). Sarkar et al. mentioned that using big data technology such as the machine learning approach performs better than the traditional statistical models, in predicting future events (17). The analysis and forecasting of the evolution of occupational accidents and injuries data are the subjects of concern for society to identify the event's impacts. The prediction trends may help the industry players to improve their existing work safety policies and introduce the best intervention solutions (18).

Companies are obligated to maintain a record of severe work-related injuries and illnesses since the establishment of workplace safety rules. These records contain text narratives that can be analyzed and represented numerically. Consequently, predictive modeling tools will be able to identify relevant scoring trends. Despite being a valuable source of information that provides a variety of facts on the occurrences of a workplace accident (19, 20), the use of text narratives has been restricted and is still insufficient (21, 22).

Therefore, this systematic review aims to synthesize recent research using text-analytic with machine and deep learning techniques to mine the occupational injury narratives. We reviewed the potential of these techniques in predicting occupational injury outcomes, identifying the gap, and proposed a framework for the novelty of this study.

This paper enhances the field of occupational injury research as the findings from the systematic review will provide insights into the potential of deploying more consistent, robust, and timely-efficient prediction algorithms for occupational injury outcomes.

This paper is organized into six sections including the introduction. In Section Methodology, a detailed description of the methodology is presented. The main findings are explained and elaborated in Section Results and further discussed in Section Discussion. Meanwhile, the conclusion and future works are recommended in Section Conclusions and future works.

## Methodology

### Search strategy

A systematic literature review was conducted to identify, assess and interpret the related studies that applied text-mining and NLP techniques in occupational injury. The search was performed by using eight databases, namely Scopus, Web of Science, Science Direct, PubMed, IEEE Explore, Emerald, MEDLINE Complete, and dblp:computer science bibliography. The search approach involved a combination of keywords which are: [(“occupational injury”) OR (“occupational accident”) AND (“natural language processing”) OR (“text mining”) OR (“injury narratives) AND (“machine learning”) OR (“deep learning”)]. All relevant references were exported to EndNote reference manager software. Additionally, the Preferred Reporting Items for Systematic Reviews and Meta-Analyses (PRISMA) was performed to meticulously identify and screen the potential articles (23).

### Inclusion and exclusion criteria

In this study, the inclusion criteria were set as all articles including open access that applied the text-mining and NLP techniques in extracting the injury narratives, classifying and predicting the occupational injury. Plus, any study that used machine and deep learning algorithms with NLP-based tools in the occupational injury area were also included. The search results were limited to original research in English and published from 2016 to 2021. The scope is bound to recent 5 years of literatures of state-of-the-art published works to ensure the reliability and the updated trends of the machine and deep learning-based NLP models included in this current study. Conference papers, journal reviews, news, editorial papers, book series, book and book chapters were excluded. The inclusion and exclusion criteria that are applied in the advanced search of the databases are described in Table 1.

**Table 1:** Inclusion and exclusion criterion.

**Criterion**	**Inclusion**	**Exclusion**
Sources	Journal/Research Article	Conference papers, journal reviews, news, editorial papers, book series, book and book chapters.
Language	English	Non-English
Period	2016 to 2021	<2016
Area	Engineering, Occupational Safety and Health, Public Health, Artificial Intelligence	Other than Engineering, Occupational Safety and Health, Public Health, Artificial Intelligence

### References selection

There are three steps involved in selecting the related articles which are the identification, screening of title and abstract, and eligibility. First, the relevant studies were identified by advanced search in each database through the subject area's keywords while following the inclusion and exclusion criteria as shown in Table 2. This resulted in 394 references retrieved in the identification phase, with 54 duplicated references were removed. Next was the screening phase. In this phase, the title and abstract were screened. Any references that did not match the inclusion criteria were excluded. There were 208 studies removed after the title screening. An additional 58 articles were excluded as the abstract did not meet the inclusion criteria. The third phase was content screening. This phase involved reviewing the articles' full text to ensure that the 38 articles were eligible to be examined.

**Table 2:** Search strings for eight databases.

**Searching texts**	**Science direct**	**Scopus**	**IEEE Xplore**	**Web of Science**	**Emerald**	**MEDLINE Complete**	**dblp**	**PubMed**
Occupational injury AND natural language processing	15	35	0	8	0	13	0	2
Occupational accident AND natural language processing	14	47	1	8	3	4	0	1
Occupational injury AND text mining	2	25	1	0	2	2	0	0
Occupational accident AND text mining	1	27	0	0	1	10	0	0
Occupational injury AND injury narratives	13	39	0	4	2	17	0	4
Occupational accident AND injury narratives	4	39	0	0	0	5	0	0
Workplace injury AND natural language processing AND machine learning	5	3	0	0	0	2	9	0
Workplace injury AND natural language processing AND deep learning	2	3	0	0	0	1	0	0
Total including duplicates	56	218	2	20	8	74	9	7
Sub-total including duplicates	394							
Total selected articles	27							

**Figure 1:**
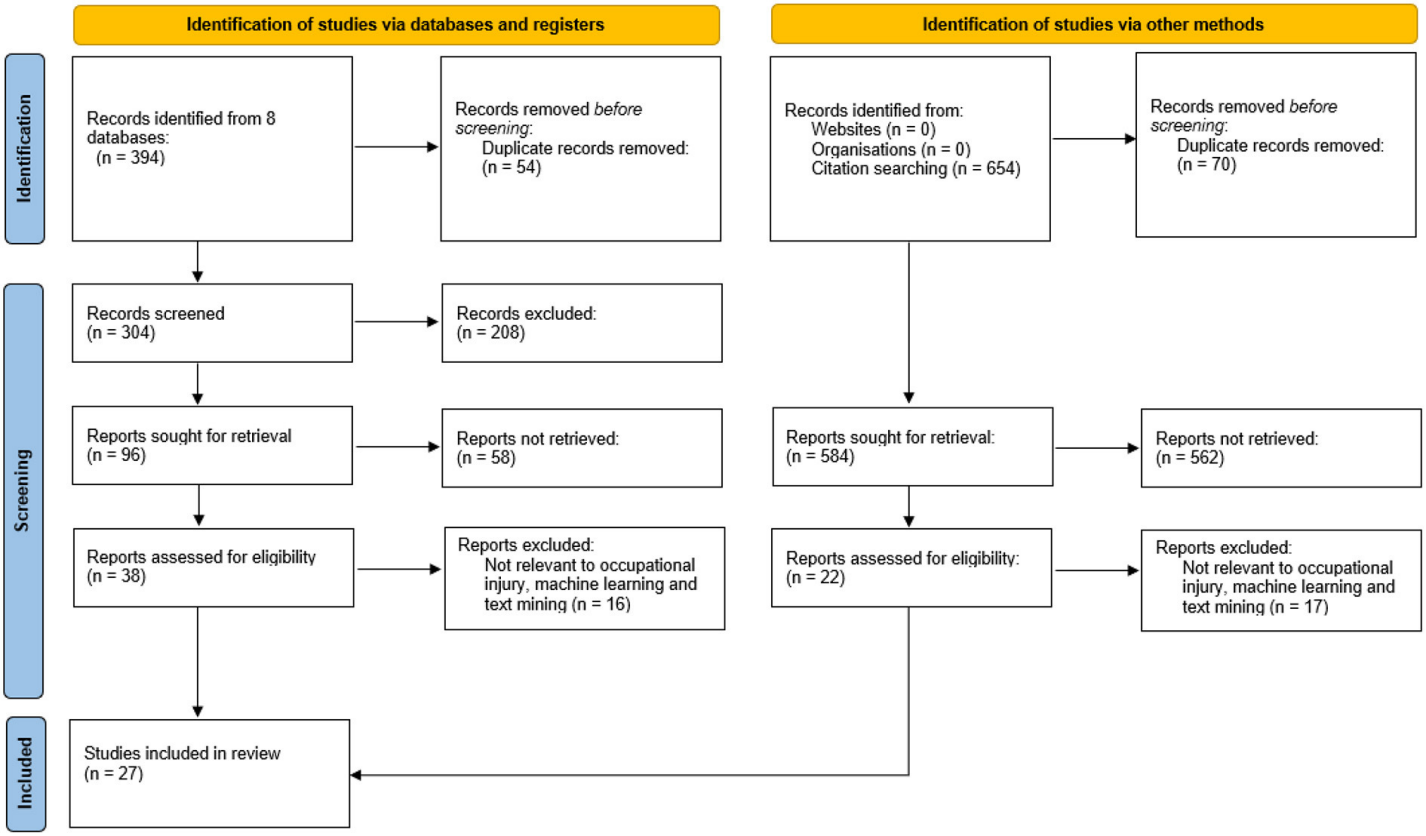
PRISMA flowchart.

The full text of the remaining articles was read thoroughly to confirm that the inclusion criteria were fulfilled and satisfied. All the important aspects such as the objectives of the papers, methodology and the findings of the studies were evaluated. Following the evaluation, 16 articles were removed due to the studies did not apply any text mining, NLP and/or NLP-based machine learning algorithms. On that account, a total of 22 articles were included for review from the database with an additional five articles selected from other methods. The details of the 27 references selection are illustrated in Figure 1 (PRISMA).

### Quality assessment and data extraction

In ensuring the quality of the review assessment, articles from the eight databases were gathered and screened independently by all authors. Before data extraction was conducted, at least two authors must agree on those references to be included. The first, second and third authors carried out the compilation of extracted information and compiled them in an organized table. The remaining authors then checked all the produced data. Any dissimilar views between authors were resolved through discussion. To systematically organized the data, data extraction table was established to include study characteristics as follows: (i) databases used in the studies; (ii) type of industries; (iii) focus of the papers; (iv) methodologies; and (v) main findings of the studies.

## Results

**Figure 2:**
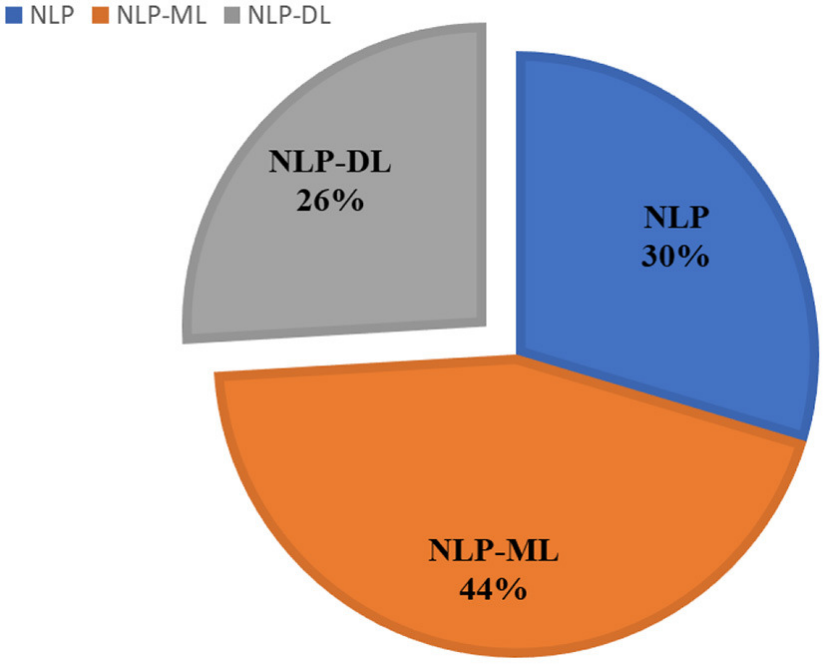
Percentage of three main algorithms for 27 articles.

**Figure 3:**
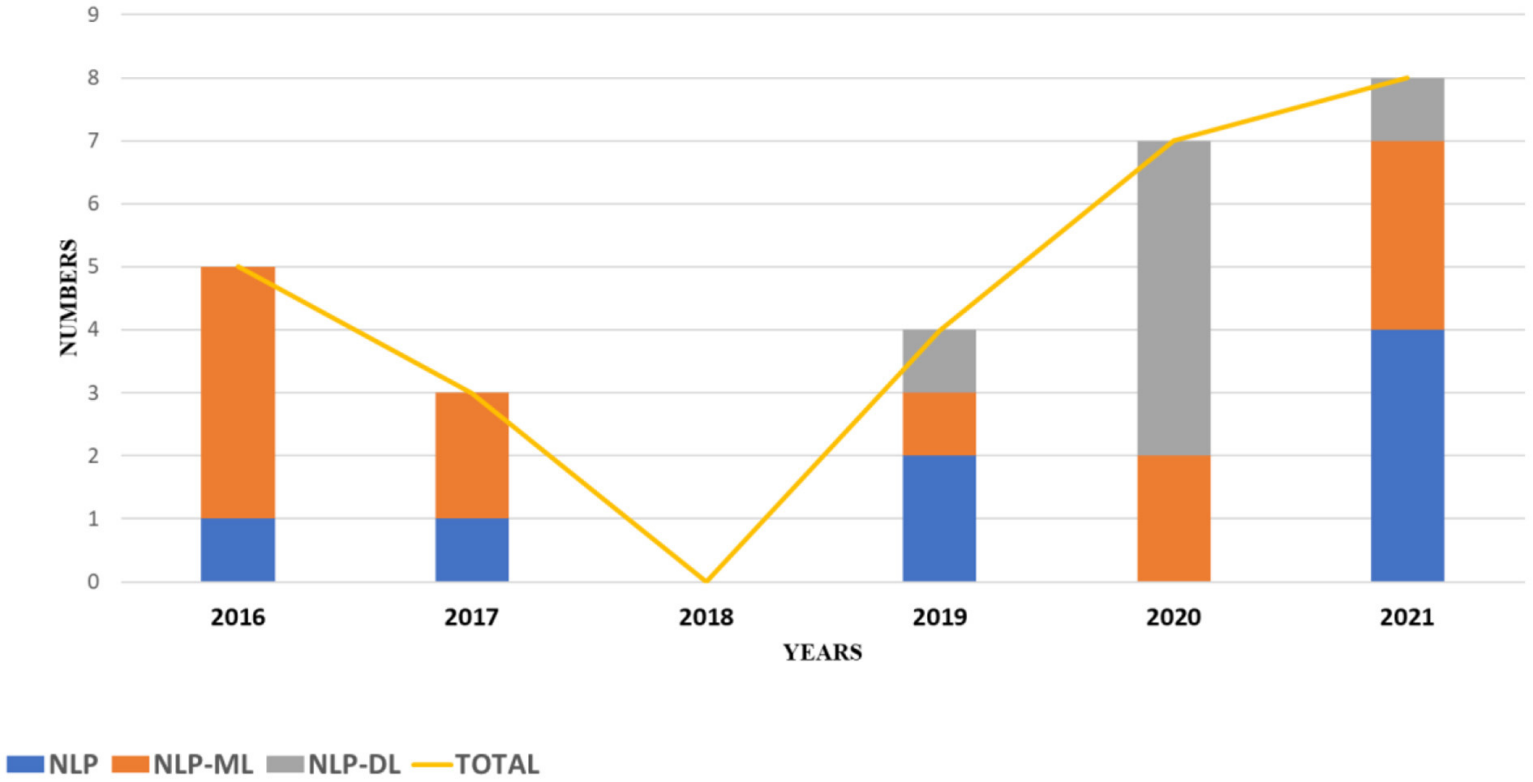
Distribution by year of publication.

Once the identification and screening process is completed, 27 articles are selected in this study. These articles are selected based on their utilization of text mining and NLP techniques in extracting significant information from the occupational injury narratives. These articles can be analyzed into three sub-groups as shown in the pie chart of Figure 2. The highest contribution to performance prediction for occupational injury outcomes with 44% (12 articles) is on machine learning-based NLP model (NLP-ML), followed by 30% (8 articles) for NLP models (NLP) and the lowest contribution of current knowledge is on deep learning-based NLP models (NLP-DL) with 26% (7 publications). The similarity of techniques applied in the reviewed literature is shown in Table 3, meanwhile, the summary of these papers is in Table 4, and distribution by publication year is illustrated in Figure 3.

**Table 3:** Similarity of techniques in existing related studies.

**Techniques**	**Articles**	**Total**
**Text mining/natural language processing** *R-language, Semantic retrieval model, Tacit knowledge extraction model, domain lexicon, Word2Vec model*	(24–31)	8
**Machine learning-based NLP** *Random Forest, K-means Clustering, Support Vector Machine, Linear regression, K-nearest Neighbor, Decision Tree, Naïve Bayes, XGBoost, Classification And Regression Tree*	(32–43)	12
**Deep learning-based NLP** *Artificial Neural Network, Convolution Neural Network, Stacked Gated Recurrent Unit, Recurrent Neural Network, LSTM*	(19, 44–49)	7

**Table 4:** Short Summary of review papers.

**References**	**Objectives**	**NLP-based approach**	**Results**	**Limitation of the study**	**Research gaps and proposed work**
Yedla et al. (19)	Identify the potential of text narratives in predicting the injury outcomes and days away from work	LR, DT, RF, and ANN	ANN had the best overall accuracy (0.78) for fixed field entries and RF had the best overall accuracy (0.94) for injury narratives.	Data imbalance problems are not considered.	Future studies can expand by using deep learning models such as CNN and RNN. The use of a Generative Adversarial Network (GAN) to overcome data imbalance problems should be explored.
Tixier et al. (30)	Extract valuable new safety knowledge from large datasets, in terms of “safety clashes”	Graph mining, Hierarchical clustering on Principal Components	Graphical features are useful in identifying the combinations of attributes.	Findings are limited to one dataset only.	Follow-up research should expand the generalizability of the methods to other occupational contexts or settings.
Nanda et al. (40)	Test the Bayesian decision support system to auto-codes large datasets	NB models; Single-Word (SW) and Two-word Sequence (TW)	TW had higher sensitivity (0.69) than SW (0.66); accuracy increased when the two models agreed (0.80)	Not include information on the nature of the injury and affected body parts.	To include the coded information on the nature of the injury and body parts in the models.
Bertke et al. (41)	Compare the performance of NB and LR models; Investigate the performance of adding TW into a single model and test the feasibility of the models with external datasets	NB and regularized LR	LR performed better than NB, accuracy (0.80), and adding TW improved the performances of both models.	Lack of quality control on the narratives.	Evaluation of a database with less descriptive narratives will likely have lower success with auto-coder.
Kim and Chi (25)	Suggests an NLP-based prototype of a Construction Accident Management System	Semantic retrieval model using Okapi BM25 and thesaurus; Tacit knowledge extraction using rule-based, conditional random field (CRF)	Retrieved results 97% relevant to the accident reports; Knowledge accuracy using rule-based (93.75%) and CRF models (84.13%)	Practical limitations in rule generation involve grammatical errors and various expressions from the injury reports.	Required more data to fully learn the tacit knowledge feature. The necessity to apply the proposed system to the real-world construction field for system optimization.
Cheng et al. (45)	Suggests a hybrid model address sequential problems in text characteristics of accident reports	DT, KNN, NB, LR. SVM, LSTM, GTU and hybrid model–SGRU	SGRU had the best overall performances (0.69)	Existence of imbalanced data distribution in the dataset.	Exploration in sequential learning models such as RNN variants. Focus on the application of data balancing techniques such as over-sampling/under-sampling to tackle the issue of imbalanced data distribution in datasets.
Liu et al. (46)	Suggests a novel framework, JUMPER understands the sequential decision process in text	JUMPER model; CNN as SentEnc and RNN as controller.	JUMPER reduced the length of text reading up to 40%; up to 30% speedup for prediction; finding key rationale up to 6%; classification, JUMPER achieved better performances on all tasks.	The inaccuracy of neural networks was fed with too much irrelevant information.	Incorporating symbolic reasoning into the output layer in a multitask setting to explicitly handle inference.
Xu et al. (28)	Provide an improved approach to extracting risk factors from accident reports	Text-mining approach–domain lexicon	Verified TF-H is favored in measuring risk factors	Limited source of text documents.	Extraction of valuable information from different text documents will be given different corpus and tasks and produce a better model.
Chokor et al. (33)	Assess the strength of unsupervised machine learning-based NLP in re-arranging the type of accidents	K-means clustering	Four accident attributes of clusters–“fall,” “struck by objects,” “electrocutions” and “trenches collapse”	Limited to only one specific geographical data.	Models can be improved by investigating a larger sample of occupational injury reports.
Goh and Ubeynarayana (35)	Evaluate various text-mining models to classify the accidents	SVM, LR, RF, KNN, DT, NB	SVM had the best average f1 score (0.62); Linear SVM with uni-gram and RF with uni-gram were the best classifiers	An excessive number of terms/features and unrelated elaboration of narratives in the reports.	Developing a more intelligent pre-processing of the narrative such as using rule-based methods may eliminate unrelated narratives to the occupational injury.
Luo et al. (26)	Suggests a text-based analytic method using fall accident cases for accident analysis	R software; Apriori algorithm	TF-IDF calculation identified 28 causal factors and six groups of accident types; the strong correlation between the causal factors (confidence level = 100%); the occurrence of an accident is the result of the synergistic effects of the causal factors.	Lack of input on the external environment such as temporal characteristics.	Expansion of detailed association analysis including the environmental factors for more comprehensive models.
Marucci-Wellman et al. (38)	Compare the human-machine approaches to classify the occupational injury narratives	SVM, LR, NB (SW and bi-gram)	LR model had the best performance (0.74); SVM-NB bi-gram models performed as the paired models (0.89); SVM-NB bi-gram-NB SW had improved performance with 0.93	Handling short noisy injury narratives in many administrative datasets.	Research on finding rare categories of occupational injury narratives shall be enhanced by the integration of NLP and ensemble approaches.
Oyedele et al. (47)	Compare the state-of-art algorithms with conventional machine learning to analyze the accident reports	R software; DNN, GBM, XGB, SVM, KNN	Deep learning outperformed boosted trees and other algorithms (0.967); GBM-XGB-DNN had better accuracy (>0.90)	Focused on one construction company.	Findings should be validated through additional research by collecting data from several organizations. Implement robust interface techniques and develop deep feedforward neural networks for holistic safety management.
Song and Suh (29)	Utilize text-mining and LOF models to detect anomalous accidents type	LOF algorithm	Prioritized major clusters–“filling related,” “detection-related,” “ventilation-related,” and “waste-related” accidents	Lack of data quality; poorly written reports on the accident sequence and insufficient keywords.	Documents containing more keywords will produce better text-analytic. Research on forecasting for preventive processes using text documents should be proposed.
Suh (27)	Identify sectoral patterns and common factors of accident processes using injury narratives	LDA algorithm; R software	Five sectoral patterns were identified; eight topics of accident factors were discovered.	Inconsistency of the data quality; poor quality of narrative text consisting of few words and usage of the single data source.	The value of big data analytics can be enhanced by using multiple data sources and incorporating other external factors related to occupational injury in data analysis.
Zhang et al. (34)	Classify the causes of accidents and identify the common objects that cause the accidents	SVM, LR, KNN, DT, NB, proposed ensemble model; Rule-based chunking approach	The proposed ensemble model with optimized weights achieved the best performance (0.68); 11 labels as the causes; 10 most common objects identified	The issue on the vagueness of natural language processing techniques.	Exploration of more advanced RNN variants and NLP frameworks such as Natural Node. Emphasize the application of data balancing techniques.
Zhong et al. (44)	Suggests the deep learning methods to extract unstructured text automatically and provide a visual presentation of accident classification	CNN, SVM, NB, KNN, LDA-based network analysis	CNN outperformed all methods (0.63); nodes with a higher sample degree of centrality were “falls” and “collapse of objects”	Focused only on construction dataset and issues relates to labeling.	Testing the algorithms on much larger samples and developing a multi-label classifier to process occupational injury texts with multiple labels.
Tixier et al. (36)	Apply RF and SGTB in predicting the injury	RF, SGTB	SGTB models reached higher predictive skills; models predicted three safety outcomes (0.236<RPSS<0.436)–“injury type.” “energy type.” “body part”	Focused only on the construction industry which limits the generalizability of the models.	More training on model stacking algorithms and using training data extracted from other sectors to widen the model application.
Sarkar et al. (39)	Develop a model to predict injury severity based on reactive and proactive data	SVM, ANN, NB, KNN, CART, RF; LDA-based topic modeling	RF outperformed other models; performances of classifiers were better in mixed data; KMSMOTE performed better in oversampling technique	Focused only on the steel industry that limits generalizability and the dataset used has limited observations.	Analysis of a larger amount of data for better generalizability of the results. Exploring the data balancing techniques such as oversampling, under-sampling, algorithm-level, or cost-sensitive. Consider including other factors as input data.
Tixier et al. (24)	Test the attributes and safety outcomes can be extracted automatically and accurately from the injury reports	R software based on hand-coded rules and keywords	R capable to scan the narratives with high recall (0.97), precision (0.95), and f1 score (0.96)	The system is not robust to erroneous input such as misspelled, missing, or unseen words.	NLP systems should be hybrid with different ML algorithms. Explore the potential of data-mining methods such as hierarchical clustering.
Baker et al. (37)	Predict the safety outcomes	RF, XGB, SVM, CART	XGB, RF, and SVM performed comparably for classification; XGB-RF models as model stacking performed better than in single model	Addressing the limitations of judgement bias with empirical data.	Utilization of more powerful predictive algorithms such as neural networks to improve human decision-making. An interesting area of research is to predict the success or failure of occupational injury occurrences.
Ganguli et al. (32)	Analyze the injury reports on public databases to be applied to private datasets	RF	With the high success of 95% classification on MHSA data; models were able to classify with about 96% accuracy in a non-MHSA data	Too dependent on the terminology and the report writing style.	Improve automation by standardization of occupational injury report writing.
Zhang (48)	Explore the state-of-art text mining techniques for the automatic classification of occupational accident reports.	Hybrid structured deep neural network with Word2Vec	Proposed neural networks outperform each baseline model in terms of weighted average f1 score with 0.723	The size of the corpus used in this study is relatively small.	Application of data balancing techniques such as oversampling when pre-processing the accident causes. Building a larger domain-specific corpus can be beneficial for improving the quality of learned word embedding.
Guanyang et al. (42)	Generate word clusters of words as contributory factors and form causal dependency.	NLP with K-means clustering and text mining techniques of co-occurrence network	Both methods are capable of identifying contributing factors. The co-occurrence network approach exhibits advantages in extracting dependency among the contributory factors, while K-means clustering is only able to indicate general correlations.	A co-occurrence network can inevitably omit important contributing factors.	Incorporating supervised learning techniques and fundamental network theory to identify underlying patterns of how the nodes (key objects) are connected.
Neththi et al. (43)	Extract sources of hazards from occupational injury reports by using Text Mining (TM) and Natural Language Processing (NLP) techniques	Rule-based extraction tool, SVM, Kernel SVM, KNN, NB, and RF	The F1 score obtained through the rule-based model is 0.95. The worker factor is the highest contributor to construction site accidents	Limited literature focusing on extracting sources of hazard in the construction industry.	Further modified and utilized to extract any other reports in various domains by adjusting the N-gram files accordingly, provided that the N-grams be enriched with relevant words and phrases
Zhong et al. (49)	Develop a novel framework that provides the ability to analyze hazard records automatically	Latent Dirichlet Allocation (LDA) model, CNN, Word Co-occurrence Network (WCN), and Word Cloud (WC)	The trained CNN-based deep learning model outperforms the shallow learning model	The complexity of the framework lies in the architecture of the CNN, especially on the hyper-parameter tuning.	Focus on determining how the integration of advanced semantic and syntactic features with the domain-specific knowledge of CNN models can result in improvements in the classification process.
Jing et al. (31)	Developed a text-mining method for chemical accident cases based on word embedding and deep learning.	word2vec model and LSTM	Trends in chemical accidents could are obtained through correlation analysis based on word embedding	Complete injury reports can be hard to obtain. Data from websites are often incomplete, and complete cases are not fully disclosed to the public.	Establish a high-quality chemical accident case dataset.

The most common focus of the reviewed articles was to propose the framework or models used in analyzing the occupational injury text data through text mining and NLP techniques. Tixier et al. emphasized the testing of text mining techniques in extracting valuable safety knowledge from large attribute datasets (30). Nanda et al. proposed a Bayesian model to auto-code large portion of datasets (40), meanwhile, Bertke et al. compared the previous models (40) with regularized Logistic Regression (LR) models (41). In 2019, Kim and Chi suggested a prototype model consisting of semantic retrieval and tacit knowledge extraction in developing the construction accident knowledge management system (25). As the techniques dynamically advanced, the researchers initiated to propose deep learning models to improve the application of these techniques (21, 45, 46). In addition, a text-mining approach through domain-lexicon (28) and a pilot study that used the fast-Text model was introduced as improved frameworks to extract the information from occupational injury reports (50).

Next, the focus of the papers was to evaluate the utilization of text-mining and NLP-based techniques in classifying occupational accidents (26, 33, 35, 38, 47). Also, these techniques were used to identify anomalous accidents from the narratives (29). Other than that, the clustering approach in classifying occupational injury was executed (51). Suh et al. used the sectoral pattern to categorize the type of occupational accident (27). Another important objective discovered from the papers is the ability of these techniques in determining the causes of occupational injury (26, 27, 34, 52). Interestingly, a paper by Zhong et al. enhanced the findings by providing a visual representation of the accident causal factors (44). As these techniques are useful in prediction analysis, several types of research were conducted to predict the occupational injury (44, 52) and severity of the occupational injury (39). Moreover, the potential consequences of the occupational injury, as well as, the outcomes of the occupational injury were also predicted (19, 24, 37, 53).

In most recent studies, the potential of occupational injury textual information to predict the days away from work was tested (19). Similarly, Maior et al. developed a specific model to determine the possibility of the employees taking an injury leave based on occupational accident (54). Likewise, there were papers focusing on exploring the proposed models or algorithms that were previously analyzed using the public domain databases, to be applied in the private sector datasets, or outside databases (32, 40).

In overall, based on reviews of the 27 articles, the proposed frameworks and models of these techniques in combination with Machine Learning (ML) or Deep Learning (DL) algorithms were proven capable to identify occupational injuries. This includes the classification of types of accidents, determining the cause of such accidents and its severity, and forecasting consequences of accidents such as days off work or injury leaves. It is also found that, the techniques are suited well with external databases.

### Text-mining and NLP-based techniques

Text-mining is defined as “the process of deriving information from text data which is not previously known and uneasy to be revealed” (55). NLP is the “techniques that involved various areas in computational linguistics, artificial intelligence, mathematics and information science” (34). One common task in text-mining is the classification of the text, meanwhile, NLP can analyze semantic and grammatical structures in the narratives.

These techniques need to undergo the pre-processing and feature extraction steps to process the textual information. The steps are; removal of upper case or lower case and punctuations from text data, stop words removal, tokenization, stemming and lemmatization, part of speech (POS) tagging, N-grams, parsing and semantic reasoning (34, 45). These steps are found to be a crucial phase to ensure that the critical information in occupational injury narratives can be captured for further testing.

Tixier et al. focused on building the NLP system to test the proposition of the attributes and safety outcomes to be automatically extracted from the unstructured reports (24). They applied the R programming language based on hand-coded rules and a dictionary of keywords to develop the automated NLP system. It has revealed that the R system can examine the unstructured information with a high precision of 0.95. Additionally, Luo et al. (26) utilized R and its software packages to propose a text mining-based method for detecting the occupational accident causal factors and types of the occupational accident based on “term frequency-inverse document frequency” (TF-IDF). Also, Suh used R in constructing the document-term matrix as the input for the “latent-Dirichlet allocation” (LDA) algorithm in the text mining approach to identify the sectoral patterns of occupational accidents from the narratives (27).

To add, Song and Suh employed the text mining algorithm to extract keywords and calculated the “local outlier factor” (LOF) to prioritize the type of bizarre occupational accidents in a chemical plant industry (29). In other studies, by Kim and Chi, the NLP system was chosen in developing a prototype of a construction accident knowledge management system (25). Both applied the semantic retrieval model and tacit knowledge extraction model which successfully analyzed the knowledge accuracy of 93.75% and 84.13% based on the rule-based and “conditional random field” (CRF), respectively. The text mining framework was further improved in research by Xu, where they executed a tailored domain lexicon to identify the safety risk factors from the occupational accident reports (28). The findings approved that based on the verified “term frequency” (TF-H) superiority, the proposed framework was able to measure the important factors from the narratives.

Currently, Macêdo and Maior had tested the NL-state-of-art method named “Bidirectional Encoder Representations from Transformers” (BERT) in their study (53, 54). Maior et al. focused on predicting the injury leave taken based on the occupational accident at a hydropower company and Macêdo used to identify the potential consequences of occupational accidents in the oil refinery industry. For both studies, the model achieved 74.4% (54), and 96.87% accuracy respectively (53). Similarly, another study explored the NLP-FastText tool to classify the documents of the occupational injury claim (50). The FastText employed in this study successfully classified the documents with high accuracy of 95.7%.

However, as natural languages are complex (45), most research in a modern NLP tool used machine learning algorithms to overcome the complexity of the natural languages.

### Machine learning approaches

Chokor et al. used K-Means, a clustering algorithm to evaluate the machine learning and NLP tools' strength in re-arranging the type of accidents (33). The findings found that the occupational accident may happen due to four factors namely; “falls,” “struck,” “electrocution” and “collapse of trenches.” Tixier et al. applied the Random Forest (RF) and Stochastic Gradient Tree Boosting (SGTB) models to predict the safety outcomes of the occupational injury (36). From the Rank Probability Skill Score (RPSS) used in their study, it revealed that the models were able to predict the “injury type”, “energy type” and “body part affected” which ranked within 0.236<RPSS<0.436. Then, the Naïve Bayes (NB) model was proposed by Nanda et al. to auto-code the cases with the accuracy of the results increased to 80% when the two NB models; Single-Word and Two-Word Sequence were fixed (40).

The algorithms were further evaluated in a study by Goh et al. that executed six machine learning models named Support Vector Machine (SVM), Logistic Regression (LR), K-Nearest Neighbors (KNN), Decision Tree (DT), RF and NB in categorizing the injury narratives from the public domain databases (35). It is found that the SVM superseded other algorithms with an average F1 score of 0.62. Next, the human-machine ensemble methods were introduced by Wellman et al. to clarify the occupational injury narratives (38). The study found that the LR model was the best performing algorithm with an overall sensitivity of 0.89, NB with SVM as the best pairing model and a triple ensemble of NB_sw_ = NB_bi−gram_ = SVM outperformed others with an overall sensitivity of 0.93, respectively. Likewise, Zhang et al. also proposed the combination of SVM, DT, KNN and NB as the ensemble model with the Sequential Quadratic Programming (SQP) algorithm acting as the weight optimizer (34). The optimized ensemble model achieved the highest F1 score of 0.68.

In 2020, Baker et al. used the NLP method to improve the findings of Tixier et al. (36) by introducing two new models; XGBoost and linear SVM, as well as model stacking (37). The two new models were performed comparably and this validated the NLP tools by Tixier et al. (24). Interestingly, by doing the model stacking, the injury severity outcome was able to be predicted as it was not discovered in the original study (36, 37).

The K-means clustering approach was used once again in 2020 by emphasizing the Silhouette analysis and Principal Component Analysis (PCA) to strengthen the models in selecting the best dimensions of each cluster (51). Sarkar et al. applied a set of six prediction tools; SVM, NB, KNN, CART, and RF including Artificial Neural Network (ANN) to predict the occupational injury severity using reactive and proactive data (39). From the results, the RF outperformed other classifiers. This study also highlighted the application of oversampling techniques to address the imbalance of data.

As per the review, the most widely used machine learning techniques are K-means clustering, NB, SVM, DT, KNN, LR and RF. Plus, the techniques are enhanced as the researchers have explored the potential to ensemble the various machine learning algorithms in a single model for a better result.

### Deep learning approaches

Deep learning techniques have been proposed as an effective approach to automatically extract features for text classification (44). Yedla et al. introduced an “Artificial Neural Network” (ANN) together with several traditional machine learning tools in their study (19). The findings showed that ANN had the best overall accuracy of 0.78 as compared to LR, DT and RF. Next, Cheng et al. proposed a hybrid model named “Symbiotic Gated Recurrent Unit” (SGRU) to address the sequential problems in text characterization of occupational injury narratives (45). The hybrid model achieved the best overall performance with an F1 score of 0.69 as compared to other machine learning models including the “Long Short-Term Memory” (LSTM). Interestingly, this study proved that the SGRU was the first RNN type with high performance used in classifying the occupational injury textual data.

Then, Liu et al. developed a novel framework known as “JUMPER,” a neural system that understands text as a sequential process (46). Also, they included the “Convolutional Neural Network” (CNN) as a sentence encoder and “Recurrent Neural Network” (RNN) as a controller that reads the input sentences in sequence. As predicted, the “JUMPER” model had better performances in all tasks, and eventually, it reduced the length of text reading up to 40% and fasten the inference up to 30% for prediction. In other research by Zhong et al., they were interested in demonstrating CNN's performances in comparison with other machine learning models, i.e., SVM, NB, and KNN in extracting the unstructured narratives (44). It was proven that the CNN model outperformed all the other models examined in their study.

Neural networks have gained interest and become a promising technique over the conventional machine learning tools as it has achieved significant improvements in these tasks, especially when dealing with sentences and text classification. Despite that, there is still an insufficient amount of research that focused on the application of deep learning in examining occupational injury reports.

## Discussion

This current systematic review paper revealed that the fundamental concern in NLP approaches is text classification. The NLP tools are too dependent on the terminology used and the style of writing in the occupational injury narratives and the description of how the workplace accident happened. This problem may cause the “vector space model” to fail in capturing the context of words or sentences used in the occupational injury narratives (32, 35, 46). It is supported by the previous systematic review paper that highlighted the importance of the consistency of terminologies, abbreviations and text normalization steps in strengthening the text classification (56).

The previous text classification was adapting the hand-crafted templates such as the “bag-of-words” features and it is based on the type of machine learning models applied for the classification. However, the features can contain some crucial information on the grammar and the order and arrangement of words. Another feature is the “n-gram” which can ease the conflicts in word or sentence representations by considering several continuous words (57). Next, the “word-to-vector” (Word2Vec) is introduced to capture the semantic information (58). This word embedding feature is trained over a huge corpus and is mostly used in neural networks (46). “Word2Vec” is currently-known as to be effective in learning the vector representation of words (31, 32). Thus, this advancement may assist to improve text classification.

Other than that, limitations of the stop words list related to the occupational injury domain and the problem with the proper annotation of POS tags, also contributed to the misclassification issues (34). In addressing these limitations, the pre-processing steps in NLP tasks shall be improved by introducing a large number of unlabeled data to explore more keywords related to the occupational injury domain. In addition, the future approach may propose a more intelligent pre-processing method for the reports for example the rule-based method to remove unnecessary elaboration in the narratives (35).

The performances of NLP will be depending on the quality and quantity of the data as the nature of text-based analytics is highly dependent on the dictionary, keywords, tokenizers and morphemes (25, 29). The extraction from various text documents will give different corpus and tasks, generating more prominent results (28).

Recent text learning tools such as BERT by Google AI (51, 53, 54) and Fast-Text by Facebook's AI Research Lab (50) are suggested to be examined in this domain. As well as, other NLP frameworks for example Natural Node, Erelsgl/limdu and Stanford NLP. It is believed that the ongoing testing and training of the NLP techniques are essential to improve the efficiency and efficacy of the automatic classification system and then, proceed to the application of other machine and deep learning methods.

Various machine learning-based NLP techniques have been constructed on shallow models such as the SVM, LR and RF trained on high dimensional and sparse features. These conventional machine learning-based NLP techniques are still efficient and able to produce the expected findings, yet, these techniques liaise heavily on hand-crafted features which are time-consuming and often partial (59). Following this concern, the NLP research is now tailored to the application of deep learning tools.

The reviewed literature found limited studies that demonstrated NLP techniques and experimented with deep learning methods. Rapid improvement of the deep learning model suggests additional investigation of the latest developed model in analyzing the attributes in the occupational injury narratives and comparing the performances with the existing proposed models (45). Deep learning is one of the branches of Artificial Intelligence (AI) which attempts to replicate the learning model of the human brain, as well as, to link between the raw text and the expected outcomes, making the deep learning models more powerful, especially the neural networks (60). Neural networks are based on the dense vector representations that have been producing greater outcomes on numerous NLP tasks (59).

The text narratives' potential to predict occupational accident outcomes can be expanded using CNN and RNN (19). CNNs are wired to capture the most important information in a sentence. They are effective due to their ability in mining the semantic clues in contextual windows, low complexity and are easy to train as the network learns throughout the optimization phases with a reduced number of parameters (49, 61). Nonetheless, the limitation of CNNs is to preserve “sequential order and model long-distance contextual information.” To further matched such type of learning is the Recurrent models (RNN). RNNs are specifically developed to be executed with “sequences” (62). The strength of the RNNs is the memorization of the results of previous computations to be used in the current computations (63).

In addition, the RNN variants are suggested as an interesting topic to be explored such as the LSTM unit and GRU (34, 45). LSTM models have been proposed for sequence-to-sequence mapping (encoder-decoder frameworks) used for machine translation and text summarization, meanwhile, GRU is chosen for its simplicity (63). However, more studies are needed to boost the effectiveness of these variants.

As the nature of occupational injury narratives is unstructured and unlabeled, thus, it requires advanced unsupervised or semi-supervised techniques. These learning structures are still in their developing stage but, the deep learning-based NLP approaches are on the right track to ensure better execution using unstructured evidence (46, 59). The main highlight of the deep learning-based NLP methods is they may provide a way to harness a huge amount of calculation and data with little engineering by hand (64).

**Figure 4:**
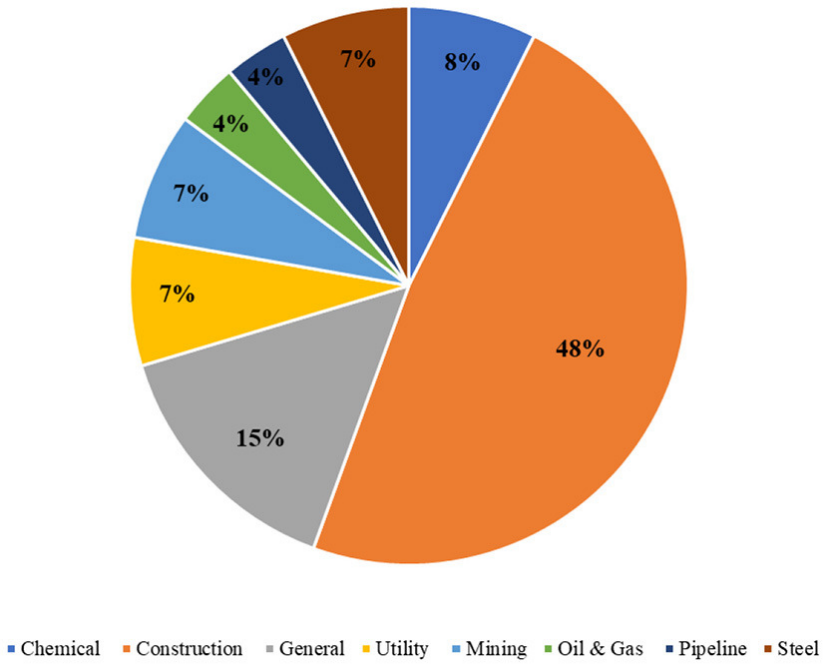
Industrial sectors in existing literature.

Next, majority of the previous studies focused on the construction industry This is due to the nature of the construction environment which is complex and uncertain (65) and this industry is labeled as the riskiest compared to others (25, 66). Unlike other industries, the nature of the construction industry is project-based and the production activity is diverse and not conducted at a single location (67). Thus, much attention is given to the construction industry. However, other industries are also present which are mining (19, 32), steel (39, 52), hydropower (54), oil and gas (37, 53), electricity (47, 51), and chemical plant (29). It was proven that the analysis of occupational injury reports is expanding across sectors. The researchers realized that the extraction of the injury narratives other than the construction industry may widen the ability to apply the proposed algorithms (36, 52). The number of studies according to the industrial sectors is illustrated in Figure 4.

Also, the analysis shall be expanded to other complex systems such as aviation and the process industry that may generate a high risk of occupational injury to improve the performance of the models (39). The proposed model in each sector shall be applied to the real-world scenario and the actual field test should be conducted to acquire feedback, especially on the user interface. This may help the researchers and practitioners to further optimize the proposed system (25).

About the databases gathered in the studies, some of the narratives are publicly accessed such as the “Occupational Safety and Health Administration (OSHA)” (27, 33–35, 44, 45), “Mine Safety and Health Administration” (19, 32), and “Survey of Occupational Injury and Illness” (40), beside the databases from the relevant government agencies (25, 26, 28). However, most of the studies represent the internal databases from the respective sector and industry, mainly the occupational injury reports including the records from preliminary hazard analysis (53) and worker's compensation claims (38, 41, 50). In a recent study (28), the data is gathered and analyzed from various sources which were including the published information by the authorities, published papers, and books. It is recommended that the value of big data analytics will be more emphasized with the representation of multiple data sources (27). In parallel, the analysis of a large amount of data and the collection of data from various organizations will help to validate the findings, thus, producing better performances (33, 39, 44, 47).

Other than that, to further improve the extraction of the information from the textual injury reports, it is best to include other external factors related to occupational injury as the input variables (27). Researchers may explore the analysis of more occupational injury characteristics, for example, the “activity-based factor,” “causal-and-effect relationship,” and “factor-and-severity relationship” to reveal more mechanisms contributed to workplace injury (28, 40, 68). Additionally, the documents on engineering and technical aspects, as well as, the environmental factors are encouraged to be incorporated in producing comprehensive information for results improvement (26, 53).

Such information discussed in this paper is highly valuable, as it can be executed to improve the existing models and enhance the knowledge to better understand, predict, and prevent occupational injury occurrences.

### Improving prediction model by using multimodal data

In the occupational injury research domain, it is common to demonstrate the machine and deep learning approaches on the numerical or categorical data inputs (32). However, the ultimate goal is to combine diverse data from several modalities, as opposed to a single modality. Using multimodal data is predicated on the notion that several modalities can provide a more complete and comprehensive view of occupational injury incidents. Therefore, realizing the advantages of multimodal data integration, we proposed a comprehensive framework, by defining multimodal features that include the structured data parameters in numerical or categorical such as socio-demographic, nature of the injury, affected parts of the body and industrial code, meanwhile, the unstructured data is extracted from the textual narratives in the occupational injury reports. These notes are extracted from the narrative reports prepared by the Safety and Health Engineer and Occupational Health Doctor. This is the highlight of using multimodal data learning since it necessitates the integration of expertise in domain knowledge and technical aspects (56, 69), enhancing the successful applications of the predictive model of occupational injury outcomes.

A significant amount of data, including multimodal data types, can be collected, handled, and analyzed using deep neural networks. It employs layered structures for data analysis (70) and offers a “hidden layer” with the ability to transform input data into valuable outputs. As they can recognize linguistic and grammatical components, “assemble” related words, and map them together, NLP-based neural networks are also designed for classification tasks. As a result, this technique may enhance the entire decision-making process and forecast performance.

**Figure 5:**
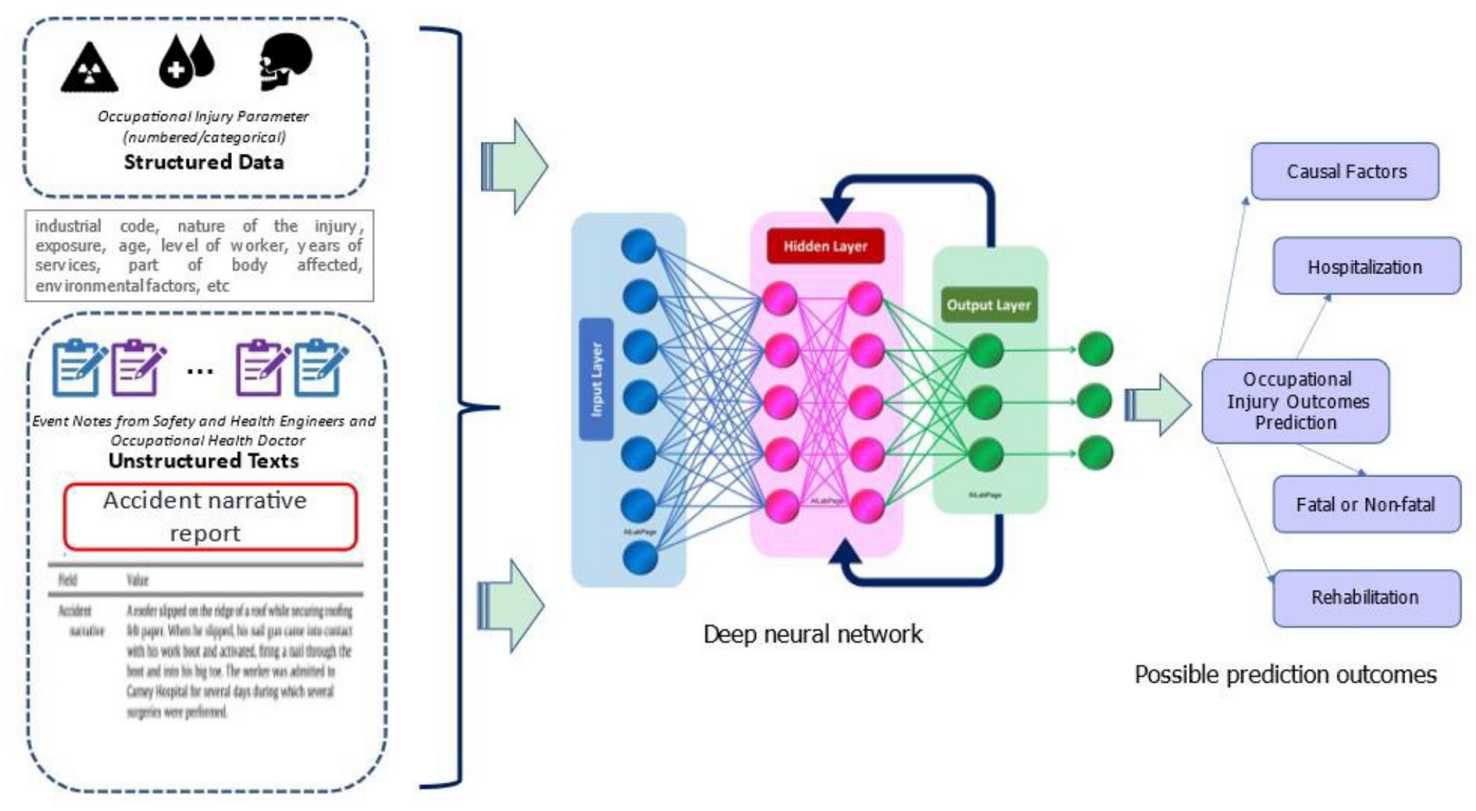
Proposed framework of multimodal prediction learning.

In occupational injuries, multimodal data and deep learning represent a potential area of study. This approach is provided for future research on the reproducibility and generalizability of prediction algorithms, as there are still limited number of cases employing this technique. The proposed structure is depicted in Figure 5.

## Conclusions and future works

Overall, this study revealed that the machine and deep learning-based NLP models such as Naïve Bayes, K-Nearest Neighbor, Support Vector Machine, Decision Tree, Logistic Regression, Random Forest, K-means and Neural Networks were potentially proven in analyzing the occupational injury narratives in terms of classifying the type of accidents, identifying the occupational accidents causal factors and predicting the occupational injury. However, it is believed that the application of these techniques is still lacking in the occupational injury research domain, especially with only seven articles utilizing the deep learning-based NLP technique.

Also, this study discussed several limitations of these techniques and was able to categorize them into three main points, (i) inconsistency of terminologies and abbreviations from various styles of injury report writing contributed to the misclassification of text, (ii) concern on the quality and quantity of the data sources shall not be confined into small samples and distinct populations as it will reduce the generalizability of the models, and (iii) issue on the existing imbalanced of data distribution in the datasets may lead to the complexity of analysis. Thus, attention is needed to overcome these challenges for the better expansion of these text analytic techniques.

We believed that this paper is the first of its type to comprehensively review the text mining techniques with the machine and deep learning methods in analyzing occupational injury narratives. As the predictive nature of neural networks is believed to be powerful, it is recommended that the extensive exploration of deep neural network-based NLP in examining the information from the occupational injury reports may enhance the existing practices in the occupational injury research domain.

## Data availability statement

The original contributions presented in the study are included in the article/supplementary material, further inquiries can be directed to the corresponding authors.

## Author contributions

MK, KH, NA, and KL developed the study protocol as well as a major contribution to the article writing. MK, MO, and KH performed the identification and screening, assessment of data eligibility and quality, and information extraction of the review articles. MAs, KS, SS, MAz, and XW checked all the synthesized data and approved the final version for publication. All authors have read and approved the manuscript.

## Funding

The research is funded by Xuzhou Science and Technology Project under Grant No. KC21182.

## Conflict of interest

The authors declare that the research was conducted in the absence of any commercial or financial relationships that could be construed as a potential conflict of interest.

## Publisher's note

All claims expressed in this article are solely those of the authors and do not necessarily represent those of their affiliated organizations, or those of the publisher, the editors and the reviewers. Any product that may be evaluated in this article, or claim that may be made by its manufacturer, is not guaranteed or endorsed by the publisher.
